# Supplementary material for: Chromothripsis during telomere crisis is independent of NHEJ, and consistent with a replicative origin
Source: Genome Res. 2019 May;29(5):737–49. doi: 10.1101/gr.240705.118 (PMC6499312; doi:10.1101/gr.240705.118)
Supplement: Supplemental Material [file supp_gr.240705.118_Supplemental_file_1.zip › contigs/annotated_contigs/DB79/contig.2.DB79_length_514_mean_cov_5.24902723735.docx]

**DB79_length_514_mean_cov_5.24902723735**

```
TATTTCCAAAACTTTTTCACCACCACCCAGAAACTCTGTAACCATGATGCAATAACCCCCGTCCTCCTCCTCCTAGCCCCTGGTAACCT
 >chr17:76844230-76844556 - E=1e-184 p=9e-03
CTAGTCTACTTTCTGTCTCTATGGATTTTCCTATTCTAAATATTCTATCATCCTGCATGATTCCACCTATGTTATGTCCAGCTCACTTT

GCACGTCTTCAAGGTCCATTGGTATTATAGCATGGATCCATGCTTCCTTCTTTTTCATGGCTAATATTCCATTGTGTGCATCTATTACA

TTTTGTTTATCCATTCATCCATCAGTAGACACTTGGGTTGCTTCCGCCTTTTGGCTATT|TGTAT|ACAGTCTCTATATATTTTCTTGA
                                                                 >chr17:76756966-767571
TAAATGGGATACCCACCTCCCGGGTTCAAGTGATTCTCCTGCCTCAGCCTCCTGAGTAGCTGGGACTACAGGCATGCACCACCACACCC
49 + E=2e-98
GGCTAATTTTTGTATTTTTTGTAGAGACAGAGTTTCAACATGTTGGTCAGGCTGGTCTCGAACTCCCGACC
```
